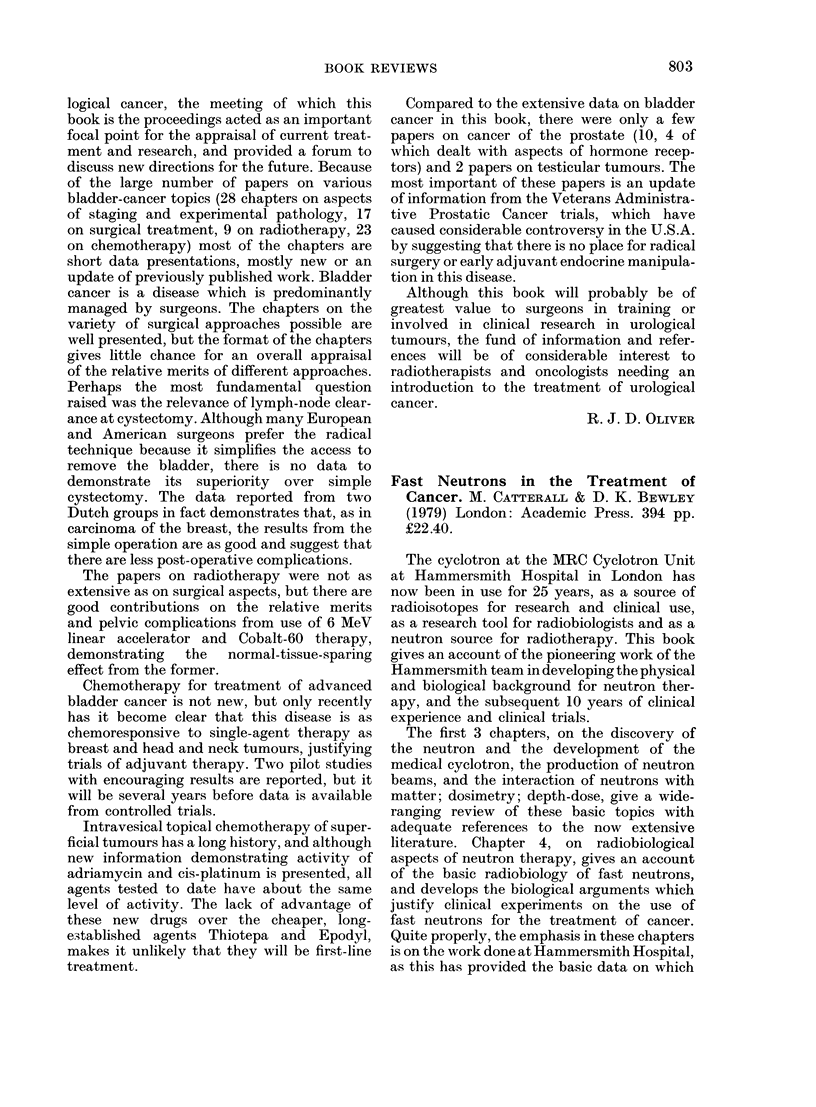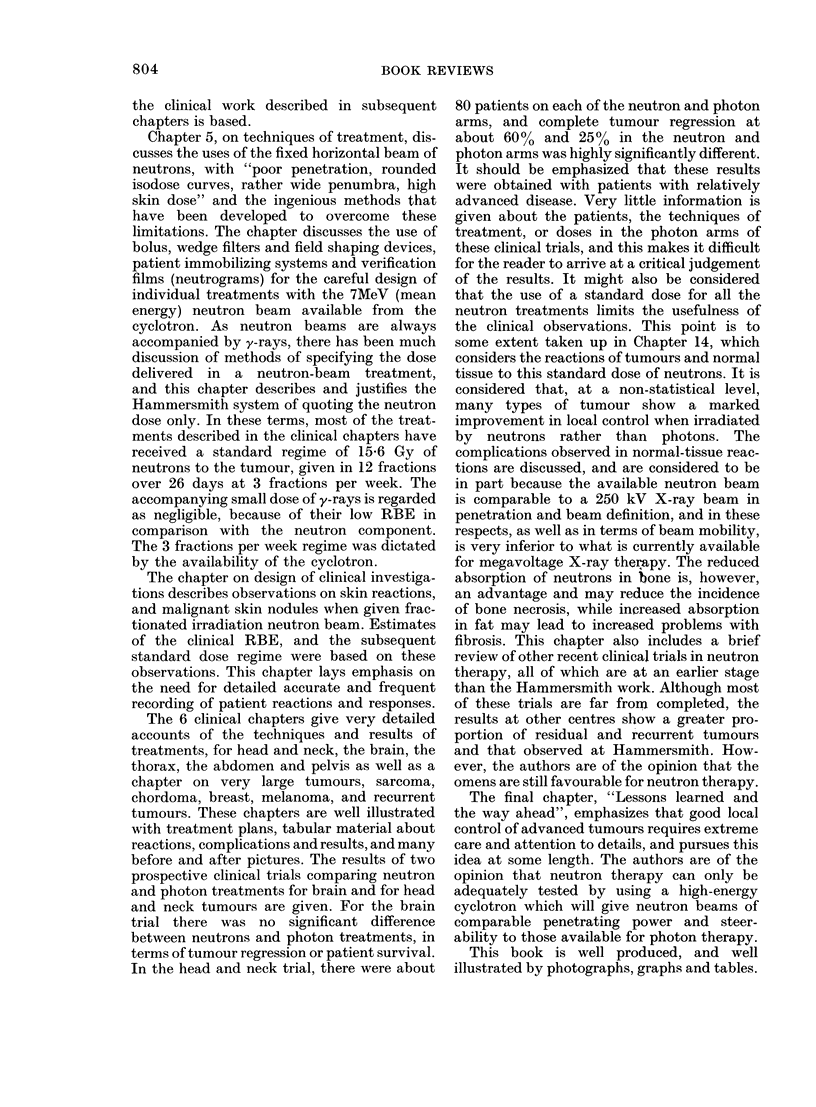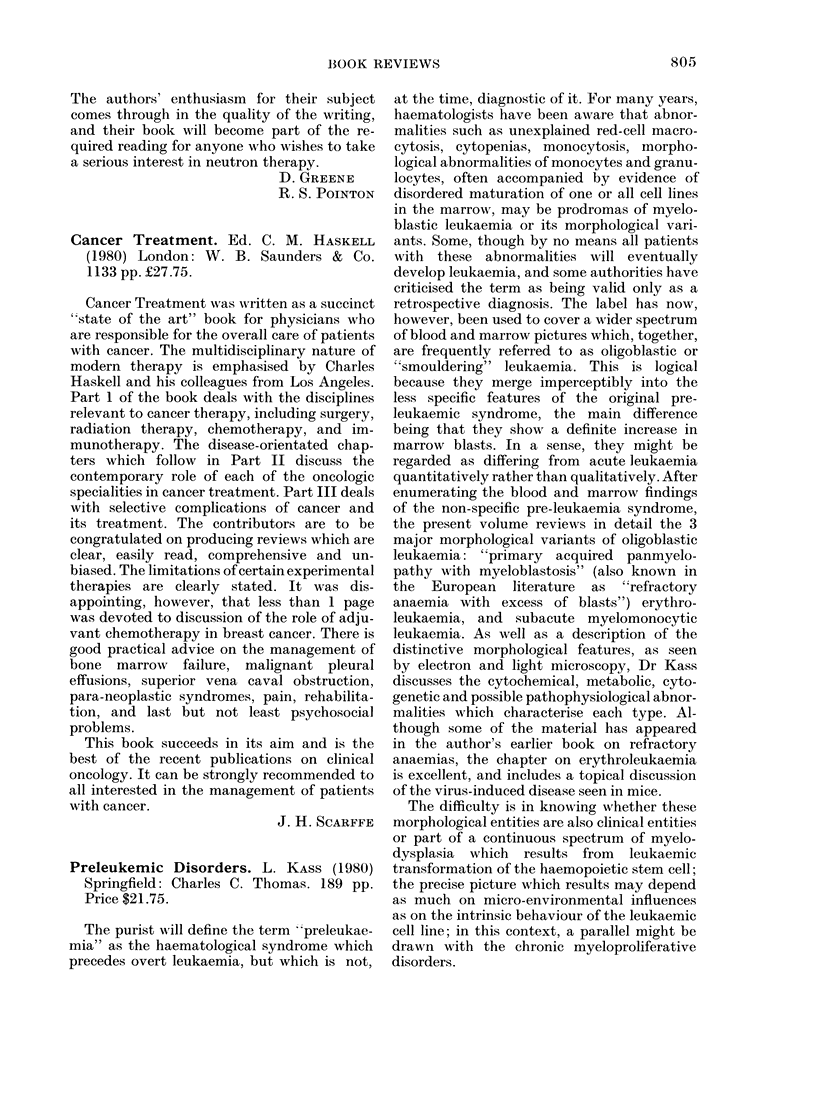# Fast Neutrons in the Treatment of Cancer

**Published:** 1980-11

**Authors:** D. Greene, R. S. Pointon


**Fast Neutrons in the Treatment of Cancer.** M. Catterall & D. K. Bewley (1979) London: Academic Press. 394 pp. £22.40.

The cyclotron at the MRC Cyclotron Unit at Hammersmith Hospital in London has now been in use for 25 years, as a source of radioisotopes for research and clinical use, as a research tool for radiobiologists and as a neutron source for radiotherapy. This book gives an account of the pioneering work of the Hammersmith team in developing the physical and biological background for neutron therapy, and the subsequent 10 years of clinical experience and clinical trials.

The first 3 chapters, on the discovery of the neutron and the development of the medical cyclotron, the production of neutron beams, and the interaction of neutrons with matter; dosimetry; depth-dose, give a wide-ranging review of these basic topics with adequate references to the now extensive literature. Chapter 4, on radiobiological aspects of neutron therapy, gives an account of the basic radiobiology of fast neutrons, and develops the biological arguments which justify clinical experiments on the use of fast neutrons for the treatment of cancer. Quite properly, the emphasis in these chapters is on the work done at Hammersmith Hospital, as this has provided the basic data on which the clinical work described in subsequent chapters is based.

Chapter 5, on techniques of treatment, discusses the uses of the fixed horizontal beam of neutrons, with "poor penetration, rounded isodose curves, rather wide penumbra, high skin dose" and the ingenious methods that have been developed to overcome these limitations. The chapter discusses the use of bolus, wedge filters and field shaping devices, patient immobilizing systems and verification films (neutrograms) for the careful design of individual treatments with the 7MeV (mean energy) neutron beam available from the cyclotron. As neutron beams are always accompanied by $\gamma$-rays, there has been much discussion of methods of specifying the dose delivered in a neutron-beam treatment, and this chapter describes and justifies the Hammersmith system of quoting the neutron dose only. In these terms, most of the treatments described in the clinical chapters have received a standard regime of 15·6 Gy of neutrons to the tumour, given in 12 fractions over 26 days at 3 fractions per week. The accompanying small dose of $\gamma$-rays is regarded as negligible, because of their low RBE in comparison with the neutron component. The 3 fractions per week regime was dictated by the availability of the cyclotron.

The chapter on design of clinical investigations describes observations on skin reactions, and malignant skin nodules when given fractionated irradiation neutron beam. Estimates of the clinical RBE, and the subsequent standard dose regime were based on these observations. This chapter lays emphasis on the need for detailed accurate and frequent recording of patient reactions and responses.

The 6 clinical chapters give very detailed accounts of the techniques and results of treatments, for head and neck, the brain, the thorax, the abdomen and pelvis as well as a chapter on very large tumours, sarcoma, chordoma, breast, melanoma, and recurrent tumours. These chapters are well illustrated with treatment plans, tabular material about reactions, complications and results, and many before and after pictures. The results of two prospective clinical trials comparing neutron and photon treatments for brain and for head and neck tumours are given. For the brain trial there was no significant difference between neutrons and photon treatments, in terms of tumour regression or patient survival. In the head and neck trial, there were about 80 patients on each of the neutron and photon arms, and complete tumour regression at about 60% and 25% in the neutron and photon arms was highly significantly different. It should be emphasized that these results were obtained with patients with relatively advanced disease. Very little information is given about the patients, the techniques of treatment, or doses in the photon arms of these clinical trials, and this makes it difficult for the reader to arrive at a critical judgement of the results. It might also be considered that the use of a standard dose for all the neutron treatments limits the usefulness of the clinical observations. This point is to some extent taken up in Chapter 14, which considers the reactions of tumours and normal tissue to this standard dose of neutrons. It is considered that, at a non-statistical level, many types of tumour show a marked improvement in local control when irradiated by neutrons rather than photons. The complications observed in normal-tissue reactions are discussed, and are considered to be in part because the available neutron beam is comparable to a 250 kV X-ray beam in penetration and beam definition, and in these respects, as well as in terms of beam mobility, is very inferior to what is currently available for megavoltage X-ray therapy. The reduced absorption of neutrons in bone is, however, an advantage and may reduce the incidence of bone necrosis, while increased absorption in fat may lead to increased problems with fibrosis. This chapter also includes a brief review of other recent clinical trials in neutron therapy, all of which are at an earlier stage than the Hammersmith work. Although most of these trials are far from completed, the results at other centres show a greater proportion of residual and recurrent tumours and that observed at Hammersmith. However, the authors are of the opinion that the omens are still favourable for neutron therapy.

The final chapter, "Lessons learned and the way ahead", emphasizes that good local control of advanced tumours requires extreme care and attention to details, and pursues this idea at some length. The authors are of the opinion that neutron therapy can only be adequately tested by using a high-energy cyclotron which will give neutron beams of comparable penetrating power and steerability to those available for photon therapy.

This book is well produced, and well illustrated by photographs, graphs and tables.

The authors' enthusiasm for their subject comes through in the quality of the writing, and their book will become part of the required reading for anyone who wishes to take a serious interest in neutron therapy.

D. GREENE
R. S. POINTON